# On Cold Water in Burns and Scalds

**Published:** 1802-11-01

**Authors:** 


### *To the Editors of the Medical and Phyſical Journal.*

GENTLEMEN,

MUCH has lately been written on the ſubject of cold ablution in fevers, a remedy of very remote date, as thoſe who are familiar with the works of the ancients well know. An hiſtorical account of the employment of cold water, internally and externally, in ſuch caſes, would be uſeful and acceptable to the medical world. *Floyer's Pſychrolusia* would afford many materials, or it might be republiſhed with notes and conſiderable additions.

Being on this ſubject, I ſhall offer an extract from a pamphlet entitled the "Curioſities of Common Water," printed about eighty years ago, relative to its external applications in burns and ſcalds, in which it has been lately extolled, under the title of a *new* remedy.

"Water, (ſays the author of the above-mentioned pamphlet) I have found, by long experience, to be of excellent uſe in burns and ſcalds *that are slight*, if the part is plunged immediately into the water, the colder the better. The pain will inſtantly be taken off, &c. If the part burnt or ſcalded cannot be dipped in water, you may apply water to it, with double linen cloths dipped therein, and new dipped as they grow warm; by which means I have cured burns and ſcalds in the face face without bliftering, when applied immediately before blifters did arife."

It is proper to remark, that this practitioner limits the application of cold water to *slight* burns and fcalds, whence it may be inferred that he had found it improper in large and violent injuries of that fort; to which, no doubt, the ftimulant applications of fpirit of wine, or oil of turpentine, are better fuited.

I am, &c.

